# Exploring the Utility of Virtual Clinics for Neurosurgical Patient Consults: Cohort Study to Assess Feasibility

**DOI:** 10.2196/69372

**Published:** 2025-08-28

**Authors:** Hassan A Khayat, Radwan Takroni, Majid Aljoghaiman, Jessy Moore, Mohamed Alhantoobi, Oscar Obiga, Marcos Ezequiel Yasuda, Bill H Wang, Almunder Algird, Kesava Reddy

**Affiliations:** 1 Division of Neurosurgery, McMaster University, 237 Barton St. East, Hamilton, ON, L8L 2X2, Canada, 1 9055212100; 2 Department of Surgery, King Faisal University, Hofuf, Al-Ahsa, Saudi Arabia

**Keywords:** virtual clinic, neurosurgery, Zoom, telehealth, consultation, neurosurgical assessment, consultation

## Abstract

**Background:**

The popularity of virtual clinics has increased in many settings, especially during and following the COVID-19 pandemic. However, their applicability in neurosurgical care remains understudied.

**Objective:**

The primary goal of this study was to assess the feasibility of conducting a larger, more definitive study at our hospital site in the future. We assessed participant enrollment rate, ability to complete a neurosurgical consult virtually, need for a third party to be present, and participant satisfaction rates. Preliminary evidence on the utility of virtual examination substitutes, compared to currently used in-person assessments, was also explored in our sample of neurosurgical patients.

**Methods:**

In this feasibility study, a cohort of neurosurgery patients, consisting of both new referrals and follow-up visits, was evaluated. Each patient participated in a virtual neurological assessment via Zoom and subsequently in an in-person assessment. Both visits were completed by the same physician. We compared clinical findings and treatment decisions (surgical vs conservative management) between the 2 settings and recorded patient satisfaction with the virtual consultation.

**Results:**

A total of 95 patients were deemed eligible for the study, and of the 52 patients contacted, 35 provided verbal consent and were enrolled. Both the virtual and in-person assessments were completed by 30 participants (86%) with an average length of 3.25 days between visits, which was within the required 2-week period outlined in the study protocol. No barriers were noted from participants (n=6; 20%) who required a third party to be present and this individual was present at both visits. Participant satisfaction rate with the virtual consults exceeded 90%. Clinical decisions were consistent between both visits in 28 cases, and in the 2 visits where decisions differed, it was noted to be a result of inconclusive findings during the virtual consultation. Comparison of individual examination components between the virtual and in-person consultations revealed exam findings to be consistent 77% of the time, and importantly, none of these discrepancies led to a change in clinical decision. No single examination component was noted to be inconsistent more than twice.

**Conclusions:**

These findings support the applicability of the proposed study design to a larger-scale project. No major obstacles or methodological challenges were encountered in achieving the goals of this feasibility study within the target timeframe. This study provides preliminary evidence to support further exploration of the use of virtual consults to help inform clinical decisions in a neurosurgical population.

## Introduction

The concept of the “virtual clinic” first emerged in orthopedic practice in 2011 as a solution to reduce patient wait times effectively [1]. Since then, this model has expanded to other specialties, including urology [2], ophthalmology [3], rehabilitation [4], and orthopedics [5,6]. Virtual clinics in neurosurgical care remain largely unexplored, with limited literature on their application in this field [7-9] and no studies that directly compare findings from virtual to in-person consultations.

The term “virtual clinic” lacks a universally accepted definition in clinical practice, leading to varied interpretations in the literature. In some instances, a virtual clinic may involve patient evaluations by nonphysician health professionals [3]. In other cases, it could refer to follow-ups conducted primarily through questionnaires [5]. In addition, telecommunication technologies, including real-time audio and video calls, are sometimes used to assess patients remotely [3]. A combination of these approaches has also been described in different clinical settings [5].

Virtual care offers several demonstrated benefits, such as cost savings and environmental advantages [2]. In Canada, virtual care has been shown to reduce wait times, enhance access, decrease missed appointments, and reduce hospital admissions due to unmanaged conditions. These efficiencies help to lower system costs and complement existing care options [10]. During the COVID-19 pandemic, virtual primary care visits in Canada surged from 4% to 60%, driven by the need to maintain isolation measures. According to the 2021 Canadian Digital Health Survey, virtual care saved Canadians CAD $5.9 (USD $4.3) billion, along with 89 million hours previously spent waiting in clinics or taking time off work to attend appointments [11].

The neurosurgery service at Hamilton Health Sciences (HHS), one of Canada’s largest health care systems with a catchment area of approximately 2.3 million people [12], has faced longstanding challenges with wait times. These challenges intensified during the pandemic, as COVID-19 restrictions further limited health care access. In response, virtual clinics emerged as a viable solution to alleviate pandemic-related backlogs and facilitate safe, contactless access to care. Within HHS, various models were implemented during the pandemic to establish virtual clinics, including a telemedicine system for neurosurgical patient follow-ups. Patients were contacted to discuss current symptoms and complaints, review recent imaging, and conduct basic examinations. From this experience, we explored several methods for assessing neurosurgical patients remotely, noting that virtual assessments could serve as an effective alternative to routine in-person visits.

This study aims to evaluate the feasibility and practicality of running a larger, more definitive study at our site in the future and to provide data that could be used for a sample size estimate. As well, we hope to assess preliminary evidence of the effectiveness of a virtual clinic model at informing a clinical decision for outpatient neurosurgical consultations compared to traditional in-person visits. Using an innovative approach, we compared each patient’s virtual and in-person assessments. To our knowledge, this methodology has not been used in neurosurgical care. The findings from this study may support the expanded use of virtual clinics within our organization, potentially attracting funding to enhance virtual care infrastructure. Ultimately, we aim to improve patient access to neurosurgical services in a high-demand, large-catchment setting while providing a foundation for conducting reliable neurological examinations in virtual formats.

## Methods

### Patient Sampling and Recruitment

This feasibility study enrolled 35 outpatients (both new and follow-up) recruited from the Hamilton General Hospital. From July 2023 to April 2024, 95 patients were identified by the treating surgeon or senior investigator as eligible for the study. Patients were considered eligible if they had been referred for a neurosurgical consult at our hospital, were of age to provide consent (18+years), had a laptop or tablet with a webcam, microphone and working internet, and had available imaging evidence of a probable neurosurgical diagnosis. Patients who did not have a working phone number on file, did not answer, or did not return messages (after 2 attempts) were not included in the reported number of eligible participants for this study. A clinical team member contacted candidates by phone to request permission for study representatives to reach out. A research team member then discussed the study with potential participants, and they were given sufficient time to make a decision about study participation and ask any questions they had.

Of the 52 patients contacted by the research team, 35 provided verbal consent to participate, and 17 were not interested in participating, canceled their neurosurgical consult appointment, or declined participation because they did not have available technology to complete the virtual consult. All patients were capable of providing their own consent. Verbal consent was obtained prior to scheduling a virtual visit via Zoom, and all participants received a signed copy of the consent form via email. Virtual Zoom calls were scheduled no more than 2 weeks prior to the patients’ in-person clinic visit. To maintain anonymity, each patient was assigned a random code once the data were collected. Each patient was assessed by a physician representative of the study team, and importantly, the same physician conducted both the virtual and in-person assessments with the goal of avoiding interrater bias. Based on our study aims and resource considerations, the sample size is considered adequate to achieve our feasibility objectives and provide valuable insights to guide the design of a future definitive trial.

### Consultative Methods

The table below (Table 1) outlines how the virtual examination was conducted through real-time video calls, using virtual substitutes for each component of the neurological examination. The table also compares these substitutes with the standard in-person examination methods. For standardization, the same examination techniques were applied to each study participant.

**Table 1.** Explanation of the virtual examination (Zoom) substitute used for each standard, in-person consultative method in a cohort of patients referred for a neurosurgical consult.

Exam component	Standard method (in-person)	Virtual substitute
Orientation	Ask location, date, and time	Same as standard
Mental status	Run MMSE^a^ questionnaire by the patient	Send the patient link to complete the exam
Extraocular muscles	Ask patient to follow object in H shape. Report diplopia	Ask the patient to look in 4 main directions (up, down, right, and left) and report any diplopia
Visual field	Confrontation test	Ask the patient to look across the room to a single point on the wall with one hand covering one eye. Move the other hand to enter the visual field through each quadrant; report visibility.
Facial sensory	Touch patient’s face with a cloth over dermatomes, ask for comparison	Show patient how to touch the same areas with a cloth or tissue for comparison via screen
Facial motor	Ask patient to mimic eye closure, blow cheeks, and give a big smile	Same
Hearing	Rub fingers next to each ear simultaneously	Same
Palate	Ask patient to say “Aaaa” and observe palate elevation	Same (ask patient to provide light source if needed)
Shoulder	Ask patient to shrug shoulders	Same
Tongue	Ask patient to protrude tongue and move side to side	Same
Pronator drift	Ask patient to outstretch arms and close eyes	Same
Upper limb motor	Physician examines each muscle group	Ask patient to move against gravity, using a 0.5 L water bottle as shown by the physician. Examine each muscle group
Lower limb motor	Physician examines each muscle group	Ask patient to stretch legs out while seated or to stand from chair; assess walking on toes and heels
Sensory	Light and sharp touch on all dermatomes	Show the patient how to touch areas with a cloth and compare sensation
Articulation	Say name, count 1‐5	Same
Nystagmus	Physician closely examines	Ask patient to look directly at the camera, changing gaze to periphery if needed
Dysmetria	Finger-to-nose test	Ask patient to alternate finger between nose and a chosen nearby point
Rapid alternating	Ask patient to tap one hand over the other with palm and dorsum	Same
Gait	Take steps across the room, turn, and return	Same

a MMSE: mini mental state examination.

#### Virtual Telemedicine Assessment

Patients were contacted through a real-time video call (via Zoom). The presence of a relative or nurse was allowed, similar to a face-to-face setting. Assistance was provided to patients as needed for certain examination maneuvers. The examination here is done through proposed techniques that enable the physician and the patient to conduct the examination maneuvers virtually. Patients can use their phones, laptops, or office personal computers as long as they have a working camera. Table 1 shows the virtual substitutes compared to the standard examination techniques.

#### Routine Face-To-Face Assessment

Following the virtual clinic assessment, each patient was seen in person at the clinic within a 2-week period.

### Study Outcomes

The primary goal of this study was to assess the feasibility of conducting a larger-scale project in the future. Secondary outcomes included evaluating: (1) whether clinical decisions (surgical vs conservative) changed following the in-person visit compared to the virtual visit, (2) patient satisfaction with the assessments, (3) consistency of clinical examination findings between the 2 visits, and (4) the distance patients traveled to the clinic.

To assess protocol feasibility, several performance metrics were observed to ensure reproducibility and effectiveness, including (1) the number of participants who were approached about the study and agreed, (2) the number of participants who completed both visits, (3) the completion of both visits within the 2-week period, (4) satisfaction rates of patients with virtual consult visits, and (5) the need for a third party to be present.

### Data Management and Analysis

Data from all study participants were collected using electronic forms via REDCap (Vanderbilt University). Each patient was assigned a unique code to deidentify their data. All patient information was kept strictly confidential and securely stored by the research team. Electronic data were password-protected and housed behind a firewall (Citrix and REDCap), with no paper-based forms used in this study. Usual progress notes from everyday clinical practice were used as reference in cases of conflict, discrepancy, or missing data during the study.

Collected data from all participants were pooled and analyzed using IBM SPSS Statistics for Windows (version 20.0; IBM Corp). Categorical variables were expressed as frequencies and percentages, while continuous variables were expressed as means and SD. Sensitivity and specificity were calculated to compare the ability of a virtual consultation on Zoom to help physicians make the correct clinical decision regarding patient outcome (ie, surgical vs conservative management). This was compared to in-person visits as the current “gold standard” for making a clinical decision. Sensitivity is interpreted as “the sensitivity of the virtual consult tool to correctly identify patients with a surgical outcome.” Specificity is interpreted as “the specificity of the virtual consult tool to correctly identify the number of patients who should undergo conservative management.”

### Ethical Considerations

This study is a single-center feasibility study that has been approved by the Hamilton Integrated Research Ethics Board (HiREB approval number 13408). This study was conducted following the ethical principles of the Declaration of Helsinki. All participants were contacted by the research coordinator, provided with the study consent form, and given time to review the form and ask any questions they had. Study procedures and the measures taken to ensure participant privacy were explained to all study participants prior to consent being obtained. All included participants provided voluntary verbal consent to the research coordinator. Following data collection, all data were anonymized using a random ID code prior to data analysis. Participants were not provided compensation for their study participation.

## Results

### Participant Demographics

**Figure 1.**
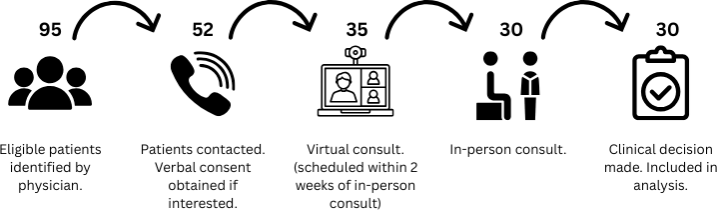
Schematic displaying the flow of patients through the study protocol and the number of individuals included at each stage of the study.

Data were collected over the study recruitment period from October 2023 to April 2024. A total of 52 patients were contacted about participating in this study, 35 patients consented and were enrolled, and all 35 attended a virtual consultation. Of these, 30 participants met with the same physician during both their virtual and in-person consultations (Figure 1). Five patients attended a virtual consultation and later a surgical clinic visit but were seen by different physicians for each and thus are excluded from the main analysis of this study.

The average age of participants was 57.32 years (±12.75 years) and 56.7% of the sample were male. Among the 30 included patients, 7 had sellar tumors, 7 had lumbar spine degeneration or stenosis, 4 had cervical radiculopathy, 3 had cranial meningiomas, 3 had gliomas, 3 had cerebellopontine angle tumors, 1 had spine tumor, 2 had idiopathic intracranial hypertension, and a sacral cyst. Of the 30 included participants, 11 were scheduled for surgery.

Virtual appointments took place via Zoom and lasted approximately 20‐25 minutes, which is comparable to the average in-person clinic visit at our hospital. The average interval between the virtual and in-person consultations was 3.25 days (minimum 1 day; maximum 5 days). Twenty-one patients (70%) were seen for new consultations and 9 patients (30%) were follow-up visits. Six participants (20%) had a third party present during both their virtual and in-person consultations (Table 2).

**Table 2.** Participant demographics and descriptive variables (N=30) in a cohort of patients referred for neurosurgical consultation at the Hamilton General Hospital between July 2023 and April 2024.

Variable	Value
Age, years, mean (SD)	57.32 (12.45)
Sex, n (%)	
Male	17 (56.7)
Female	13 (43.3)
Visit type, n (%)	
New	21 (70)
Follow-up	9 (30)
Third party present, n (%)	
Yes	6 (20)
No	24 (80)
Discrepancy in findings, n (%)	
Yes	7 (23.3)
No	23 (76.7)
Number of discrepancies, n (%)	
0	23 (76.6)
1‐3	6 (20.0)
3‐6	0
>6	1 (3.4)
Distance traveled, n (%)	
0‐20 Km	10 (33.3)
21‐40kmm	4 (13.3)
41‐60 km	10 (33.3)
61‐80 km	4 (13.3)
>100 km	2 (6.7)
Patient satisfaction (%), mean (SD)	91.1 (11.8)

Approximately one-third of the sample lived within 20 km of the clinic, while 53.4% resided more than 40 km away, including 2 participants who traveled over 100 km. Overall satisfaction with the virtual consultations was high, with a mean satisfaction score of 91.1% (±11.8%) when participants were asked “how satisfied were you with using a Zoom appointment for your visit on a scale of 0‐100” (Table 2).

Of the 30 patients recruited, only 2 (7%) were noted to have a change in the treatment plan between the virtual and in-person visits, and this was noted to be a result of inconclusive findings during the virtual consultation. The treatment decision was consistent across both visits for the remaining participants (28/30; 93%). Comparisons of neurosurgical examination components between the virtual and in-person consultations revealed that 5 patients had 1 discrepancy between findings, 1 patient had 2 discrepancies, and 1 patient had 7 discrepancies (Table 2). Importantly, none of these discrepancies led to a change in clinical decision.

The virtual consult demonstrated a sensitivity of 90% and a specificity of 98% when compared to the in-person consult, the current gold standard, for making a clinical decision for neurosurgical patients. In addition, the positive predictive value was 78% and the negative predictive value was 99%.

Upon examining individual examination components across both consultative methods, no single component accounted for more than 2 discrepancies. Discrepancies were noted in visual acuity, lower limb motor function, sensory body assessments, and gait for 2 participants, although not all were observed in the same individuals (Table 3). Differences in ophthalmologic examinations were observed but were comparatively minor. None of these differences in findings led to changes in clinical decisions by the physician.

**Table 3.** Comparison of findings for each examination component between in-person consults and virtual consults on Zoom in a cohort of patients referred to a neurosurgeon at the Hamilton General Hospital.

Examination component	Virtual consult	In-person consult	Difference noted
Orientation	0	0	0
Mental status	1	1	0
Extraocular muscles	2	1	1
Visual field	5	4	1
Visual acuity	7	8	1
Facial sensory	2	2	0
Facial motor	1	1	0
Hearing	3	3	0
Palate movement	1	0	1
Shoulder	0	0	0
Tongue	0	0	0
Pronator Drift	0	0	0
Upper limb motor	5	4	1
Lower limb motor	5	3	2
Sensory whole body	9	7	2
Nystagmus	0	0	0
Dysmetria	1	1	0
Rapid alternating	1	1	0
Gait	2	4	2

In Table 3, values refer to the number of patients with a reported finding that was abnormal for each examination component.

## Discussion

### Principal Findings and Comparison With Previous Works

This study assessed the feasibility of conducting the proposed study design in a larger-scale, more definitive trial. The design, ultimately, was structured to assess the utility of virtual consultations as an alternative to in-person consultations for neurosurgical patients, with in-person assessments currently used as the gold standard. To maintain consistency, the same health care provider conducted both assessments for each patient, allowing for direct comparisons in terms of treatment decisions (surgical vs conservative management) and clinical findings. The study findings indicate that the proposed protocol was implemented without significant barriers and met the designated timeframe (8‐10 months). Of the 52 eligible patients that were successfully contacted, 35 were enrolled in the study, giving an enrollment rate of 67%. We feel this is a satisfactory rate given the number of physicians that were involved and supports the feasibility to run a larger study at our site. Furthermore, 86% of patients completed both virtual and in-person visits and were included in our final analysis, and 100% of patients were able to be scheduled for both visits within a 2-week window. Overall, 20% of participants required a third party to be present during both consults, but this led to no barriers.

A review of the literature reveals that this particular methodology—comparing virtual and in-person consultations in neurosurgery—has not been previously applied. Several studies conducted during the COVID-19 pandemic assessing a neurosurgical population have shown high levels of satisfaction with telemedicine consultations among both patients and surgeons [7-9]. One study highlighted the perceived benefits of telemedicine from both perspectives [9]. Patients found virtual consultations to offer enhanced efficiency and improved communication, while identifying safety concerns, as well as technological and administrative issues, as drawbacks. Neurosurgeons, on the other hand, noted the benefits of increased efficiency and reduced COVID-19 exposure, while the drawbacks included challenges with physical examinations, technological issues, and concerns for patients. It was suggested that virtual consultations may be particularly suitable for postoperative reviews and discussing scan results, while new patient consultations, neuro-oncology cases, and patients with new-onset neurological deficits might be contraindicated for virtual assessments [9]. Nonetheless, in line with Chaganty et al (2023), virtual consultations may not benefit the entire neurosurgical patient population, and each patient should be evaluated individually. Importantly, to our knowledge, no other studies have directly compared virtual consultations with in-person consultations within the same patient population.

In a related study by Mahmoud et al [13], the applicability of telemedicine was prospectively evaluated in pediatric perioperative care against a historical cohort of in-person assessments. This study concluded that telemedicine could effectively bridge the communication gap between patients and physicians, with a satisfaction rate of 94% [13], close to our reported rate of 91%. While numerous studies highlight the benefits of virtual care, including cost-effectiveness, reduced wait times, and decreased no-show rates, challenges remain. Implementing a virtual care program requires advanced technical capabilities, cybersecurity, and regulatory compliance [14, 15].

Most studies on virtual care focus on metrics such as cost, efficiency, and accessibility rather than on the safety, consistency, and reliability of clinical assessments. A potential reason for this gap is the lack of tools specifically designed to evaluate these aspects. Conducting a thorough clinical examination remotely presents unique challenges, especially in neurosurgery, where traditional assessments rely on direct physical contact between patient and provider, which can significantly impact treatment decisions. These challenges hinder the broader implementation of virtual care in neurosurgical practice.

One of the main challenges we encountered in this study was developing a consistent neurological examination protocol for telemedicine. To address this, we created “virtual substitutes” for each component of the neurological examination. With these substitutes, a comprehensive neurological examination could be adapted to the virtual setting. Patients were also asked to complete previously validated questionnaires that assess specific neural functions. Following the virtual assessment, patients underwent an in-person visit, allowing for a direct comparison between the 2 modes. Based on the results of this study, the virtual substitutes we used yielded findings that were consistent 77% of the time with in-person examinations. In a future larger, more definitive study, we plan to evaluate the safety and reliability of the virtual clinic for neurosurgical consultations.

Data analysis revealed that in 93% of cases, the treating physician upheld the same treatment decision and plan (operative vs conservative management) across both virtual and in-person assessments. It is important to note that in the 2 cases (7%) where the treatment decision varied, it was a result of inconclusive findings during the virtual consultation. Both of these participants had cervical radiculopathy, and while conclusions cannot be drawn directly from this feasibility study, this may represent a diagnosis that is not appropriate to assess virtually. Clinical examination findings were consistent in 77% of cases, and no discrepancies in examination findings led to a change in clinical decision. To our knowledge, this is the first study to directly compare virtual and in-person consultations in neurosurgical patients, demonstrating a sensitivity of 90% and specificity of 98% for virtual consultations as a clinical decision-making tool in relation to in-person assessments. We report these findings with caution, acknowledging that this is a feasibility study with a small sample size. Nevertheless, we felt it was important to report these preliminary findings which support a future study to explore the use of virtual visits in neurosurgical consultations.

More than 50% of the patients in our study resided farther than 40 km from the hospital. Offering a broader range of patients, the option for virtual neurosurgical consultations could contribute to reduced carbon emissions from transportation and may alleviate the burden of travel time and costs for patients. Assuming an estimated 251 g of CO₂ are emitted per kilometer by light-duty vehicles in Canada (based on 2017‐2021 models) [16], and considering only the 16 participants who live more than 40 km from Hamilton General Hospital, the use of virtual consultations in place of in-person visits could have potentially reduced CO₂ emissions by at least 421,680 g. This represents a preliminary estimate and highlights a possible area for further exploration. Previous research highlights several demonstrated benefits of virtual care including cost savings, environmental advantages, reduced patient wait times, enhanced access to care, fewer missed appointments, and fewer hospital admissions due to unmanaged conditions [2,10,11]. Future research should aim to develop guidelines outlining which patient populations are suitable for virtual consultations and identify scenarios where this approach may not be feasible or safe.

### Limitations

The limitations of this study include the small sample size and the lack of prior validation for the virtual examination method. With a larger sample size, future research aims to validate the virtual examination methods used in this study and assess the safety and reliability of virtual consults in a neurosurgical population. More specific insights could be made regarding appropriate patient populations for virtual consults, and a larger study may provide more generalizable results. Conclusions from this study cannot be generalized to all virtual consults or to all neurosurgical patients, and while we aimed to minimize interrater bias by having the same physician meet with each participant both virtually and in person, a future study may include a subset of patients who are seen by a different physician at each visit to explore the potential influence of intrarater bias.

### Expected Outcome

Most existing literature highlights the cost-effectiveness [4,10] and efficiency [11,12] of virtual care. While the primary aim of this study was to assess feasibility at our site, it also offers preliminary evidence on the potential utility of virtual examination substitutes in the assessment of neurosurgical patients. Although the reported sensitivity and specificity should be interpreted with caution, they nonetheless provide meaningful insight into the effectiveness of these virtual tools in informing clinical decision-making. The data generated from this project contribute to the growing body of literature and can assist stakeholders in directing funding and resources toward improving access to care through safe and reliable virtual modalities. Our findings support the need for a larger study to evaluate the safety and reliability of virtual consultations and to produce more generalizable results. Ultimately, the overarching goal of this research is to enhance access to care and explore whether virtual consultations can serve as a viable alternative for neurosurgical patients.

### Conclusions

Collaborative efforts and a well-organized research team with clear objectives and timelines enabled the successful execution of this feasibility study. No major obstacles were encountered, and the project adhered to the predetermined timeframe. Preliminary data from this study show that it may be feasible to plan a larger future study and provide the data to perform a power analysis for such a study. We examined the consistency in clinical assessments and treatment plans and showed that no discrepancies in examination components between virtual and in-person consultations led to a change in clinical decision. A more statistically robust study is required to substantiate these findings and provide more generalizable results for a population of neurosurgical patients.

## Abbreviations

HHS: Hamilton Health Sciences
HiREB: Hamilton Integrated Research Ethics Board
